# Advance care planning in progressive neurological diseases: lessons from ALS

**DOI:** 10.1186/s12904-019-0433-6

**Published:** 2019-06-13

**Authors:** Antje A. Seeber, A. Jeannette Pols, Albert Hijdra, Hepke F. Grupstra, Dick L. Willems, Marianne de Visser

**Affiliations:** 1 Department of Neurology, Amsterdam University Medical Center, Academic Medical Center, University of Amsterdam, P.O. Box 22660, 1100 DD Amsterdam, The Netherlands; 2 Section of Medical Ethics, Department of General Practice, Amsterdam University Medical Center, Academic Medical Centre, University of Amsterdam, P.O. Box 22660, Amsterdam, The Netherlands; 3 Department of Rehabilitation, Amsterdam University Medical Center, Academic Medical Center, University of Amsterdam, P.O. Box 22660, Amsterdam, The Netherlands

**Keywords:** Advance care planning, Chronic progressive neurological disease, Supportive and palliative care, Quality of life

## Abstract

**Background:**

There is increasing awareness of the need for an integrated palliative care approach in chronic progressive neurological diseases. Advance care planning (ACP) is an integral part of this approach. As a systematically organized and ongoing communication process about patients’ values, goals and preferences regarding medical care during serious and chronic illness, ACP aims to involve patients in decision-making before they become cognitively and communicatively incapable. However, it remains underutilized in daily neurological practice except for speciality clinics such as ALS centers. Our aim was to study ACP in the tertiary ALS center Amsterdam and to investigate patients’ reflections on it. Subsequently we used this knowledge to formulate recommendations for integration of ACP in the care of patients with other chronic progressive neurological diseases.

**Methods:**

Non-participating observations of all appointments of patients with amyotrophic lateral sclerosis (ALS) or progressive muscular atrophy (PMA) with the treating physician, in various stages of disease, during 6 consecutive months, followed by single in-depth interviews, and an inductive analysis.

**Results:**

Twenty-eight Dutch patients participated, varying in age, gender, disease onset and severity of physical decline. ACP started directly when the diagnosis was given, by means of a general outlook on the future with progressive disability and immediate introduction to a customized multidisciplinary team. During follow-up ACP was realized by regular appointments in which monitoring of the patient’s status and clear communication strategies formed the basis of tailor-made discussions on treatment options. Patients accepted this policy as careful professional guidance.

**Conclusions:**

ACP is a professional communication process throughout the *whole* course of progressive disease. It is feasible to integrate ACP into follow-up of patients with ALS and PMA from diagnosis onwards. Supported by recent literature, we argue that such a well-structured approach would also enhance the quality of care and life of patients with other chronic progressive neurological diseases.

**Electronic supplementary material:**

The online version of this article (10.1186/s12904-019-0433-6) contains supplementary material, which is available to authorized users.

## Background

Chronic progressive neurological diseases (CPNDs) are associated with increasing disability and shortened life expectancy [1]. Patients with conditions such as amyotrophic lateral sclerosis (ALS), high grade glioma (HGG), multiple sclerosis (MS), Parkinson’s disease (PD) and other movement disorders, post-stroke status with disability, and dementia syndromes share a host of physical, emotional, and existential problems, and unmet care needs which require an integrated palliative care approach [2–6]. Advance care planning (ACP) is an integral part of this approach. It can be summarized as systematically organized and ongoing ‘process of communication to ensure that patients receive medical care that is consistent with their values, goals and preferences during serious and chronic illness’ [7]. Its goal is involvement of patients in decision-making on (future) care before these patients become cognitively and communicatively incapable to do so [8, 9].

However, ACP is underutilized in both non-neurological and neurological diseases [3, 10–13]. This may result in under- and overtreatment and poor quality of life and dying [14–17]. The underutilization appears to be mainly due to misconceptions about and unfamiliarity with palliative care, including the concept and goal of ACP [18–22].

In the Netherlands’ ALS tertiary center (NAC), ACP is commonly practiced. The rapid progression of ALS and progressive muscular atrophy (PMA) tolerates no delay in discussing personal values, life goals, and preferences regarding (future) medical care. In ALS, 50% of patients die within 36 months after symptom onset and in PMA within 48 months [23, 24]. Death is usually caused by respiratory failure as a result of progressive muscle weakness [25]. The only currently available disease-modifying drugs, riluzole, prolongs life by only a few months. The added value of edavarone is still a matter of debate [26, 27]. Supportive interventions including feeding via a gastrostomy tube (percutaneous endoscopic gastrostomy, PEG) and non-invasive ventilation do not prevent severe and progressive disability either [28, 29]. However, they are associated with prolonged survival and/or improved health-related quality of life and therefore recommended to be offered to patients with MND, in a timely manner [4, 30, 31].

We set out to explore how ACP is realized in this special setting. Our main aims were (1) to evaluate timing and content of discussions on supportive treatment options and end-of-life care and to investigate patients’ reflections on this practice, and (2) to formulate recommendations about integration of ACP in the care of patients with other CPNDs.

## Methods

We performed an inductive content analysis of observations and interviews and present our data according to the Consolidated Criteria for Reporting Qualitative Research (COREQ, Additional file 1: Table S1 [32]).

### Setting

In the tertiary ALS center of the Academic Medical Center of Amsterdam (AMC) the bad news is broken in a two-tiered appointment: a neurologist specialised in motor neuron diseases (MNDs) delivers the bad news and discusses the details of the diagnosis and its implications during a follow-up visit 2 weeks later [22]. At that very time follow-up by a multidisciplinary team specialised in MND is initiated via an appointment with the team leader, a specialized rehabilitation physician. During regular three-monthly follow-up, commonly lasting an hour, the lead of the team takes the recent history including evaluation of paramedical support and scores the actual ALS-Functional Rating Scale. The physical examination includes measurement of weight and assessment of lung function by means of slow vital capacity (VC, a measure of breathing).

### Study design

All participants were followed during a 6 months’ period by means of non-participating observations by the first author (AAS) of all appointments with the specialist in charge, either from breaking the bad news onwards, at relatively early stages of disease (group 1; seen first by the neurologist and subsequently by the rehabilitation physician during the observation period) or during more advanced stages of disease (group 2; seen by the rehabilitation physician during the observation period). The in-depth interview with every participant took place within several weeks after the observation period of 6 months.

### Participants

We included adult patients with ALS or PMA, seeking broad variation concerning the characteristics ‘age’, ‘gender’, ‘site of disease onset’, ‘severity of disease’ and ‘rate of physical decline’ (purposive sampling [33]). We considered these features to be important and rather easily accessible variables which influence patients’ experiences and reflections and thus contribute to the heterogeneity of the sample. Patients needed to be fluent in Dutch. Speech impairment was no exclusion criterion as long as communication remained possible (e.g. with the help of a speech computer). The only exclusion criterion was frontotemporal dementia. Eligible patients were first approached by either the neurologist (group 1) or the specialised nurse of the multidisciplinary MND team (group 2), and were informed about the study. When the patient showed interest, he/she was asked for verbal consent to be contacted by the first author (AAS). She would give more detailed information about the study, verbally and in writing, and ask patients for written informed consent. Patients knew that they could withdraw from the study at any moment, without explanation. Furthermore, it was guaranteed that no information obtained during the interview would be shared with anyone of the treating medical team*.*

### Ethics approval

All patients gave written informed consent. Dutch law specifies that ethics approval is only needed when ‘participants are subject to procedures or are required to follow rules of behaviour’ (https://english.ccmo.nl/investigators/legal-framework-for-medical-scientific-research/your-research-is-it-subject-to-the-wmo-or-not). As this was not the case, the approval of the local research ethics committee (REC) was waived, as confirmed in writing (14th June 2010). Participants were offered contact with the researcher or a member of their ALS team if participation in the study led to any questions or concerns.

### Data collection

The first author (AAS) collected the data between August 2011 and November 2012. During the observations of the appointments of patients with ALS/PMA and their MND specialist (to which we will refer as ‘observations’) she took extensive field notes which she worked out immediately afterwards. The second author (AJP) read all field notes and marked and labelled emerging themes, as did the first author. After reaching consensus about important themes, the first author explored and validated these during the observations which followed. For the interviews, the first and second author used an iterative method too, adapting and accentuating the semi-structured interview guide, first developed based on key concepts identified in the literature and during the observations. Furthermore, the interviewer added questions concerning each interviewed patient’s personal illness trajectory to the individual patient’s topics list. All interviews took place at the patients’ preferred location and time.

### Analysis

Observations were recorded and anonymized, interviews were audio-recorded, typed out verbatim, and anonymized by the first author who saved all original data on an external hard disc. Observation reports and transcripts were analyzed using MAXqda version 10 software [34]. The interviewer coded characteristics of the appointments and patients’ opinions concerning these characteristics, such as ‘general outlook on the future’ and ‘rituals during regular follow-up’. This was done shortly after each observation and interview, so that findings could be iteratively fed into the evolving coding tree and interview guide.

To ensure that the data analysis would accurately reflect reality, the second author validated the whole analytic process by reading all transcripts and took an active part in (sub)coding as described above. The first author (AAS) also discussed the codes with research colleagues from multiple backgrounds involved in other qualitative studies (investigator triangulation [35]). The whole process ended when saturation was reached, i.e. when no new categories or variations were needed to address the main research questions. The lists of transcripts under each code served for further analysis following the qualitative approach of empirical ethics [36].

## Results

Ten patients were observed from disclosure of the diagnosis onwards (group 1), 18 patients during later stages of follow-up (group 2). Twenty-one of these 28 patients were interviewed (Table 1), the seven remaining patients were no longer able to participate since they were too ill to communicate (6) or had died (1; Additional file 2: Table S2).

**Table 1:** Characteristics of interviewed patients

Number of interviewees	with ALS/PMA	16/5
Gender	male/female	13/8
Age (range 36–83 years)	< 55 years	7
	55–65 years	9
	> 65 years	5
Disease onset	limb/bulbar	17/4
Functional stage (when interviewed)	ALS-FRS ≥ 40 (including dysarthria^a^)	5 (1)
	ALS-FRS 31–39 (including dysarthria^a^)	8 (2)
	ALS-FRS ≤ 30 (including dysarthria^a^)	8 (4)

*ALS* Amyotrophic lateral sclerosis, *PMA* Progressive muscular atrophy, *ALS-FRS* ALS functional rating scale (maximum score: 48; a higher score represents better function retention); ^a^ moderate or severe dysarthria

All 21 interviewed patients were born and raised in the Netherlands. Sixteen interviews were held in the presence of the patients’ partners or other relatives who actively took part in the conversation. All interviews took place at the patients’ home and lasted between 45 and 120 min. Patients with (severe) impairment of speech used a chalkboard, a laptop or a speech computer. After 16 interviews no evidently new information regarding our main research questions emerged and after an additional five interviews thematic saturation was reached. No patient or relative voiced any concern about the study during or after participation.

### In the beginning

Advance care planning started as soon as the diagnosis ALS or PMA was disclosed. During two appointments within 2 weeks (two-tiered bad news appointment) the neurologist gave a rather general outlook on the future with progressive physical decline, care needs and supportive treatment options [22]. The rehabilitation physician with MND-expertise who directly took over further follow-up, repeated the information once again integrating it in a more concrete outlook on the regular consultations to follow in the outpatient clinic.

*There are many things which we cannot predict, but what we do know is that the disease has a constant progression. There will be no sudden surprises,* e.g. *total paralysis within a couple of days. (Observation patient 3, second appointment with the MND neurologist)*

*We will monitor your weight and the strength of your breathing muscles. You will be asked to stand on the scales and blow into this device (spirometer) during each check-up. Eating and breathing are the most vulnerable functions. We can assist these functions if problems arise, but in order to do so we need to know if a problem is developing. (Observation patient 4, first appointment with the MND rehabilitation physician)*

According to many interviewees, it was crucial that this general outlook on possible future care needs and options was embedded in the information on the ongoing support which would be provided by the multidisciplinary ALS team.

*It helped me, quite confused as I was at that time, to learn that there was a team that would help me, support me, people with a plan… and, of course, knowledge about the disease... (Interview patient 13)*

If patients suffered from potentially life-threatening symptoms by the time of diagnosis, such as severe shortness of breath or imminent choking, both the MND neurologist and the rehabilitation physician would directly provide detailed information about possible treatment options to enable decision-making without delay.

*I am very worried about your shortness of breath. You mentioned that you can only sleep at night in a sitting position. I am referring you directly to the pulmonology department to see how exhausted the respiratory muscles are. After that we can talk about the possibility of assisting these muscles. I am afraid we will have to make some important decisions today; do you think you are up to this? (Observation patient 10, first appointment with the MND neurologist)*

This policy appeared to fit with the patients’ tendency to ask detailed information mostly about actual disabling complaints.

Nearly all patients used the first appointments to address the issue ‘treatment wishes for the end of life’. Some patients immediately provided fully filled-in living wills and patient testaments. During the observations, we noticed that only patients raised the issue of ‘euthanasia’. Discussing the content and meaning of the – documented – wishes often revealed patients’ profound fear to choke or to stay alive in a miserable condition, and also patients’ hope to stay in control of any future scenario by documenting their preferences.

*I immediately discussed the euthanasia declaration with my general practitioner. I wanted to know whether she would help me. Otherwise, I had had to look for another GP... I have a feeling of peace now that this [euthanasia declaration] is in place. (Interview patient 5)*

Nineteen of the participating 28 patients (68%) signed a euthanasia statement. Eighteen did so within the first weeks after consultation of their general practitioner (GP), the remaining one within 6 months after diagnosis. Twenty-one of the participating 28 patients have passed away in the meantime. Fifteen of them had signed the statements, and five of them eventually requested euthanasia (23% of the deceased, 33% of those who had signed an euthanasia declaration) (Additional file 2: Table S2).

The rehabilitation physician encouraged patients to think about treatment preferences, including advance directives (AD) and support at the end of life, and to discuss these with significant others and the general practitioner. However, he did not consistently discuss these issues as long as patients did not have potentially life-threatening symptoms. With regard to decision-making on artificial nutrition and ventilation support he emphasized that it was important to keep on discussing these throughout the disease trajectory.

*It is important for me to hear how you feel about certain modalities of treatment. However, there are some decisions which you cannot easily make in advance, they can only be made when the time comes. I do not think it would be useful to decide now that you do not wish, under any circumstances, to be given any assistance in breathing should you experience shortness of breath. (Observation patient 15, follow-up appointment with MND rehabilitation physician)*

*Let’s agree to discuss your advance directive again in a couple of months’ time. I want to make sure that I stay up to date with your wishes. (Observation patient 27, follow-up appointment with MND rehabilitation physician)*

Interviewees often talked about the ‘natural’ way in which both MND neurologist and rehabilitation physician directly integrated information on possible physical decline, care needs and supportive treatment options in the consultations. Several of them explicitly stated that it made them feel at ease and enabled them to raise awkward or very personal issues and fears about the future.

*The neurologist immediately said that the team would arrange for me to stay at home if that was my wish. He had no preconceived plan of his own, and he did not think my questions were ridiculous…. (Interview patient 12)*

### During the illness trajectory

During the three-monthly follow-up visits, the recent patient history was taken and the functional status was measured by the ALS-FRS. At each visit physical examination was performed including monitoring of weight and VC. If changes in one or more of these parameters indicated evident physical decline, the MND rehabilitation physician took the initiative to discuss supportive treatment options in more detail.

As an example: in a patient with decreasing VC (between 100 and 70% of normal), the MND rehabilitation physician first gave information about the different treatment options, i.e., ‘non-invasive’, ‘invasive’ and ‘no ventilatory support’, and asked if the patient had already thought about his preferences. During the next appointment he advised consultation of a specialist in a home care ventilation centre when he learned that the patient was interested in ventilatory support. After that consultation, he reflected on the advice of the home care ventilation centre and the patient’s considerations and decision. He applied the same step-by-step approach concerning other supportive treatment options such as ‘PEG’ and care equipment options such as ‘triple chair’.

*You are spending all day trying to consume enough calories. From what you say there is barely time left for anything else. During your last visit we briefly discussed the possibility of a ‘PEG’ feeding tube. How do you feel about this, have you had a chance to think it over? Would you like to receive more information about it from our gastro-enterologist, who would place the tube? (Observation patient 18, follow-up appointment with MND rehabilitation physician)*

*Walking has deteriorated further, I stumble more often and that makes me very unsure, but I really want to keep walking my dog! - Have you considered whether using a wheelchair for outdoor activities would be an option? You see, it would take some time before it was delivered and perhaps it would just gather dust in the cellar, but it would be there should you want to use it to walk the dog… (Observation patient 11, follow-up appointment with MND rehabilitation physician)*

Most patients made it clear that *detailed* information about supportive medical treatment options was only experienced as truly meaningful when it fitted with the perception of their own physical and psychosocial condition. A few patients expressed their annoyance if in their opinion the information was untimely.

*I had problems with swallowing, but could still eat reasonably well. Discuss energy drinks with a dietician? Absolutely not! But now, I am quite happy with her [dietician] advice… Did you know that there are energy drinks which are quite palatable? (Interview patient 3)*

*I wanted to have everything arranged immediately. On reflection perhaps it would have been better if they had not tried to address all my concerns. A wheelchair was delivered to the house straight away …. Not very pleasant when you do not need it yet. (Interview patient 14)*

In line with that, the patients usually initiated discussions about supportive treatment options in relation to experienced physical decline, and tended to ask for more detailed information when physical restraints started interfering with their daily habits and way of life. Additionally, social events such as birthdays, Christmas, New Year’s Eve, but also illness and death of significant others, made patients reflect on their future with the ongoing disease.

*I refer to it as ‘looking just beyond the horizon together’. That is what I can cope with and that is what we do together during the consultation. Looking beyond that is too painful (…) Looking further would also mean that I cannot really talk about myself (….) If you can still walk short distances, it is perhaps reassuring to know that you can at some point order a wheelchair. But it is not until you are no longer able to support your head that you are keen to know which headrests are compatible with your wheelchair. (Interview patient 7)*

The MND rehabilitation physician always discussed resuscitation orders by the time respiratory problems arose since they predict weaning difficulties after a potential resuscitation with intubation. The majority of the participants had filled out advance directives (ADs) by that time.

Once set on the agenda, the MND rehabilitation physician addressed both supportive treatment options and ADs regularly. That could be due to further physical decline requiring very concrete information and definite decisions or due to time passing by requiring an update of the patient’s actual treatment preferences.

Patients were always advised to keep an up-to-date written document with them, and (changes of) patients’ preferences regarding future and end-of-life care were always reported to the GPs.

Some patients did not want to think ahead at all. In these cases, the physician asked permission to at least *once* talk about concrete treatment options when physical deterioration became prominent. He justified that by stating that ‘a good healthcare professional should provide patients with sufficient information to enable them to make well-informed treatment decisions’.

*I feel that I am obliged to know what your true wishes are, particularly in order to prevent things from happening which you do not want. Am I correct in thinking that you find it difficult to discuss such things [Do Not Resuscitate]? Shall we agree that I will come back to the subject in 6 months’ time? And that I will let you know when I am going to do this? (Observation patient 26, follow-up appointment with the MND rehabilitation physician)*

For most patients, the step-by-step approach to tailor-made supportive medical care, including a regular update on advance directives, appeared to work well. Last but not least, the anticipatory policy of the whole ALS team enabled them to become experts themselves who knew when to raise alarm.

*I notice that I get tired more quickly during the day, even if I’m only fumbling around at home. But I am not worried that my breathing is compromised. I do not have a headache when I wake up. And my vital capacity is also good. She (specialized nurse of the outpatient clinic) has just measured it, and I still score 110%. (Observation patient 1, follow-up appointment with the MND rehabilitation physician)*

However, this policy did not prevent that two of the participating patients were admitted to the emergency department in respiratory failure without a clear treatment plan. One patient had procrastinated for too long, and one patient had an unexpectedly rapidly progressive decline. The latter was intubated against her earlier expressed will as she had no (actual) advance directive present when she abruptly became dyspnoeic.

Furthermore, interviewees acknowledged that the visits to the office became more confronting as their condition worsened. For example, learning that the VC had further decreased and thus decision-making about ventilatory support could not be postponed anymore could be terrifying. Nevertheless, none of the patients could think of an alternative, or a ‘better’ solution.

*At the beginning you think ‘yes, everything is fine’, but at a certain moment the lung function does start to deteriorate and you know that of course. At the outpatient clinic you are given the hard facts, I always need some time to recover from that… (Interview patient 21)*

*It is important to stay in contact with someone who knows who you are and what you stand for, even if you can no longer express yourself fully. (Interview patient 20)*

The interviewees said that they understood why issues about supportive medical treatment options, including the end-of-life, were regularly put on the agenda. Several interviewees explicitly stated that it was the doctor’s duty to carefully check for emerging (physical) problems and have discussions about the implications.

## Discussion

The policy of the Dutch ALS outpatient clinic is to initiate advance care planning (ACP) directly when the diagnosis is delivered. On that occasion, a general outlook on the future with progressive diseases and probable care needs is given by the specialized neurologist, and an introduction to a customized multidisciplinary support team at short notice is organized. During follow-up, ACP is realized by regular appointments in which both monitoring of the patient’s status quo and clear communication strategies form the basis of iterative and tailor-made discussions on future supportive treatment. Patients appeared to accept this policy as part of the MND specialists’ professional guidance throughout the illness trajectory.

ACP as realized in the studied tertiary ALS center complies with current international recommendations on the management of MNDs. These include (1) a palliative care approach early in the course of disease, including (2) ongoing open communication and clear identification of important issues related to (end-of-life) decision-making, (3) a calm environment where time is allowed for reflection and integration of choices according to the patient’s priorities and life plans [4, 14, 37]. Importantly, recent studies show that even in ALS, the ‘paradigm [disease] for palliative care in neurology’, proper, i.e. broad implementation of these recommendations has not yet been achieved [38, 39]. In line with that our data show that ACP ‘by the book’ demands a very well-organized and dedicated care team. Still, we argue that our empirical data enable us to formulate some pragmatic recommendations for early integration of ACP in the follow-up of patients with other CPNDs.

### ACP – from diagnosis of any progressive chronic neurological disease onwards

We argue that ACP as an ongoing communication process about patients’ goals and treatment preferences should start as soon as the diagnosis of any chronic progressive neurological disease is given. There is a lack of empirical data on patients’ preferences regarding exact timing and detailedness of prognosis and treatment information. However, recent studies indicate the need of patients with e.g. HGG, MS, PD for proactive dissemination of information, education and (psychosocial) support [40–45]. Moreover, patients expect their physicians to initiate these discussions [41, 46–48]. The rather long and often unpredictable course of diseases such as MS and PD does not imply that patients have less information and support needs. In addition, at the time of diagnosis, patients with these diseases face a similar crisis as patients who are confronted with the diagnosis ALS or cancer [49–51]. In general, patients are overwhelmed by the bad news, need time to take in the medical information and to reflect on it. Follow-up at short notice appears to be helpful in establishing a better understanding of what the diagnosis ‘incurable disease’ means in the individual context [52]. In addition, it can help in establishing a communication basis for long-term follow-up, as it minimizes patients’ feelings of abandonment [22, 53]. Another compelling argument to directly start ACP is the fact that patients with various CPNDs may face impairment of cognition and hence decision-making capacity from early stages of disease onwards [54–59].

### ACP – during long-term follow-up

Our data indicate that the clearly structured follow-up of patients with MND facilitates maintenance of ACP. Our participants counted on the regular check-up of their overall health status and knew that (imminent) deterioration would lead to more detailed discussions about future supportive treatment options. Therefore, we recommend a similar regular assessment of patients with any other progressive chronic neurological disease. This assessment should address both disease-specific and commonly occurring disease-related symptoms such as pain, fatigue and cognitive problems. The latter problems can also occur early in the course of e.g. MS, PD and gliomas and have been found to be among the most function and quality-of-life limiting [48, 60, 61]. As we have shown and will discuss in more detail in the next paragraph, regular assessment would facilitate ongoing considerations and discussions about end-of-life wishes including documentation in ADs. In line with recent literature, our study indicates that ACP empowers patients to face the progression of disease. Timely information about their own health status in the context of knowledge on prognosis and upcoming supportive treatment decisions seems to make patients less anxious [62–64].

### ACP – a professional skill

Our data contributes to the concept of ACP in terms of concrete, routinely used communication strategies. Always ‘setting the agenda’ facilitated preparing the biannual update on earlier discussed advanced directives (ADs) during the next appointment. It made readdressing the topic less delicate for both parties. Furthermore, the iterative evaluation of actual wishes supported the realization of well-considered care trajectories [15, 65]. The titration of information to patients’ actual health status and symptom burden, called ‘looking just beyond the horizon’ by one of the patients, allowed the specialist to stay close to how the individual patient perceived his or her situation. It also made exchange of medical information meaningful and facilitated tailor-made decisions. It has been shown before that titration of medical information and support fits well with the ongoing adaptation to deteriorating abilities of patients with ALS [52, 66, 67]. As discussed above, we recommend that tailor-made exchange of information is equally needed for patients with other CPNDs [3, 48, 68].

ACP is a fundamental palliative care skill, just like communicating bad news and assessing (non-)motor symptom [3]. Recent literature suggests that the majority of physicians lack these skills [53, 69–71]. Yet, there is growing evidence that teaching programs do improve physicians’ communication strategies and overall satisfaction with handling difficult disease-related topics [72–76]. Thus, ongoing professional training in both iterative assessment of patients’ health status and supportive care needs and communication (strategies) is required in dealing with patients with CPNDs. Mastering these skills will facilitate ACP as part of good long-term care for patients with these diseases.

### Strengths and limitations

One strength of our study is that we observed at least three outpatient clinic appointments of all 28 participating patients in different stages of disease and that we interviewed 21 of them in depth. Participants were of different ages, gender, and had different illness trajectories. This all led to a wealth of data with consistent conclusions concerning ACP and participants’ experiences. As only one of the patients declined participation in the study, a significant selection bias is unlikely. Since interviewer and interviewees were acquainted with each other by the time the interview took place and patients’ carers were allowed to attend the interview, patients may have felt comfortable to openly and critically talk about their experiences.

A limitation of our study is that it took place in a single tertiary referral centre located in the Netherlands, where end-of-life considerations, including hastened death, are openly discussed. Most patients who were observed and all patients who were interviewed have been born and raised in the Netherlands. Most observations took place during the follow-up visits which were done by the only MND rehabilitation physician of the team and the specialised nurse. The role of other (para)medics and of the caregivers was not evaluated. Neuropsychological tests were not performed and the carers were not asked about the patient’s behaviour, leaving (subtle) cognitive and behavioural changes undetected. However, we did rule out frank dementia. Acquaintance with the interviewer and presence of patients’ carers during the interview might also have elicited socially desirable answers. Furthermore, one of the authors is involved in the patient care at the centre.

There is few empirical data on ACP in actual practice. The approach of the tertiary ALS center Amsterdam appears to be feasible from diagnosis onwards. However, we are aware of the challenges to realize ACP for patients with other CPNDs. There are important questions about the organization of these patients’ long-term follow-up, in particular the responsibilities of the involved health care professionals for timely assessment and treatment of palliative care needs. Furthermore, there is a wide variation in individual illness trajectories amongst various CPNDs which requires both disease-specific and in the individual patient health-status related support in daily clinical practice [2]. Patients with PD or MS, for example, will have very different information and support needs during successful treatment with disease-specific and immune-modulating treatment as compared to late stages of diseases when non-disease specific symptom alleviation will be more and more challenging [41, 77, 78]. We also noticed that in the presented approach there is room for improvement. The MND rehabilitation physician did regularly stimulate discussions about ADs such as do-not-resuscitate orders, but he did not readdress the euthanasia statements which patients provided. These were, without exception, discussed with the GP, interestingly mostly within the very first weeks after the diagnosis was given. This may well reflect the need to (re)gain control about the disease process including death at a time when patients expect to be totally out of control, but further research on this topic is needed [79]. Thirty-three percent of all participating patients who had signed that statement eventually requested euthanasia and died at home. The latter - counselling on the preferred place of dying - was another topic which could have been more specifically addressed.

## Conclusion

Our data contributes to increasing awareness that ACP is feasible from the ALS diagnosis onwards and may well be implemented in the care of patients with other CPNDs. We have shown that ACP is more than a distinct activity to make well-informed decisions on future treatment options and end-of-life issues [21]. ACP is a distinctive feature of good care and requires professional skills.

## Additional files

Additional file 1: **Table S1.** 32-item checklist of the COREQ. (DOCX 15 kb)

Additional file 2: **Table S2.** Characteristics of all Dutch participants. (DOC 78 kb)

## Data Availability

The dataset generated and analyzed during the current study are not publicly available. They are in Dutch language and available from the corresponding author on request.

## Abbreviations

ACP: Advanced care planning
AD: Advance directive
ALS: Amyotrophic lateral sclerosis
CPND: Chronic progressive neurological disease
GP: General practitioner
HGG: High grade glioma
MND: Motor neuron disease
MS: Multiple sclerosis
NAC: Netherlands’ ALS tertiary center
PD: Parkinson’s disease
PEG: Percutaneous endoscopic gastrostomy
PMA: Progressive muscular atrophy
VC: Vital capacity
