# Supplementary material for: Advance care planning in progressive neurological diseases: lessons from ALS
Source: BMC Palliat Care. 2019 Jun 13;18:50. doi: 10.1186/s12904-019-0433-6 (PMC6567602; doi:10.1186/s12904-019-0433-6)
Supplement: Supplementary file 1 — Table S1. 32-item checklist of the COREQ. (DOCX 15 kb) [file 12904_2019_433_MOESM1_ESM.docx]

Table S1 32-item checklist of the COREQ

| **Domain 1: Research team and reflexivity** | |
| --- | --- |
| Personal characteristics |  |
| 1. Interviewer/facilitator | 1^st^ author |
| 2. Credentials | PhD student and MD (neurologist) |
| 3. Occupation | during the study: PhD student (PhD research project ‘a new care model in neurology: palliative care in chronic neurologic disease’; not explicitly mentioned in the manuscript) |
| 4. Gender | female (not mentioned in the manuscript) |
| 5. Experience and training | experienced in performing semi-structured interviews with neurologists and in non-participating observations during the pilot of this study (as PhD); experienced in care for patients with MND (as MD; not mentioned in the manuscript) |
| Relationship with participants |  |
| 6. Relationship established | no relationship with participants was established prior to the study (section ‘participants’) |
| 7. Participant knowledge of the interviewer | participants knew about the occupation of the researcher and the goal of both the PhD research project and the study in which they took part (section ‘participants’) |
| 8. Interviewer characteristics | the researcher(s) assumed to be able to evaluate a care approach which was established to better serve the needs of patients with MND (see ‘introduction’; not explicitly mentioned in the manuscript) |
| **Domain 2: study design** | |
| Theoretical framework |  |
| 9. Methodological orientation and theory | inductive content analysis based on the ‘grounded theory’ (building new theories from the data), rounded up by an empirical ethics approach (deriving important aspects/values of the studied practice from the opinions of the interviewed patients; section ‘analysis’) |
| Participant selection |  |
| 10. Sampling | purposive (section ‘participants’) |
| 11. Method of approach | eligible patients were approached by a member of the NAC outpatient clinic Amsterdam, either the neurologist or the specialised nurse of the multidisciplinary team. When interested in participation, the patients was asked for verbal consent to be contacted by the 1^st^ author. She would give more detailed information about the study, verbally and in writing. (section ‘participants’) |
| 12. Sample size | 10 patients were followed from the bad news onwards, at relatively early stages of disease, 18 patients were followed during more advanced stages of disease; 21 patients (of the 28 who were followed by means of non-participating observations, were interviewed (table 1 and table S2) |
| 13. Non-participation | one patient declined participation when contacted by telephone (reasons: had participated in 2 other studies before and was actually suffering from recurrent pulmonary infections; table S2) |
| Setting |  |
| 14. Setting of data collection | non-participating observations: ALS tertiary centre Amsterdam; semi-structured interviews: at patient’s home (section ‘study design’) |
| 15. Presence of non-participants | semi-structured interviews: 16 patients were interviewed in the presence of the partner or next of kin |
| 16. Description of sample | see table 1 and table S2 |
| Data collection |  |
| 17. Interview guide | yes; example of the semi-structured interview guide not provided in the method section |
| 18. Repeated interviews | every patient was interviewed only once, amongst others to not interfere with his/her actual patient care |
| 19. Audio/visual recording | notes were taken during non-participating observations and developed into extensive field notes directly afterwards; the interviews were audio taped and verbatim typed out (section ‘analysis’) |
| 20. Field notes | see #19 |
| 21. Duration | duration of interviews: 45-120 minutes |
| 22. Data saturation | concerning the main research questions data saturation was reached (section ‘analysis’) |
| 23. Transcripts returned | none of the participants desired to read the transcript of their interview (not mentioned in the manuscript) |
| **Domain 3: analysis and findings** |  |
| Data analysis |  |
| 24. Number of data coders | 2 coders (AAS and AJP; section ‘data collection’ and ‘analysis’) |
| 25. Description of coding tree | yes, an example are given (section ‘analysis’) |
| 26. Derivation of themes | yes, but not described in the manuscript |
| 27. Software | MAXqda version 10 (Ref 34) |
| 28. Participant checking | no, however member checking (and triangulation; section ‘analysis’) |
| Reporting |  |
| 29. Quotations presented | yes |
| 30. Data and findings consistent | see argumentation discussion, appraisal up to the reviewers |
| 31. Clarity of major themes |  |
| 32. Clarity of minor themes |  |
