# Supplementary material for: Advance care planning in progressive neurological diseases: lessons from ALS
Source: BMC Palliat Care. 2019 Jun 13;18:50. doi: 10.1186/s12904-019-0433-6 (PMC6567602; doi:10.1186/s12904-019-0433-6)
Supplement: Supplementary file 2 — Table S2. Characteristics of all Dutch participants. (DOC 78 kb) [file 12904_2019_433_MOESM2_ESM.doc]

**Table S2:** Characteristics of all Dutch participants

| **Participant** | **Sex** | **Age**  years,  at inclusion | **Diagnosis** | **ALS-FRS** at inclusion | **ALS-FRS**  6 months after inclusion | **Main physical limitations**  6 months after inclusion  (at time of possible interview) | **Observation two-tiered appointment** | **In-depth interview** | **Euthanasia request**  signed (yes/no),  carried out (yes/no)  update data: medio 2017 |
| --- | --- | --- | --- | --- | --- | --- | --- | --- | --- |
| 1 | f | 61 | PMA, bulbar onset | 43 | 38 | dropped head and dysphagia | yes | yes | no,  no |
| 2 | m | 60 | ALS, bulbar onset | 45 | 40 | laryngospasm, dysarthria and dysphagia | yes | yes,  together with spouse and son | yes,  no |
| 3 | m | 74 | ALS, limb onset | 42 | 39 | weakness of arms, dysarthria and dysphagia | yes | yes | yes,  no |
| 4 | f | 65 | ALS, bulbar onset | 45 | 41 | dysarthria and dysphagia | yes | no,  died unexpectedly due to respiratory failure before the interview could take place | yes,  no |
| 5 | m | 64 | ALS, limb onset | 43 | 30 | weakness of all limbs and respiratory muscles | yes | yes,  together with spouse | yes,  no |
| 6 | m | 62 | ALS, limb onset | 48 | 42 | weakness of legs | yes | yes,  together with spouse | yes,  still alive |
| 7 | f | 82 | PMA, limb onset | 40 | 39 | weakness of legs and right arm | yes | yes,  together with spouse | no,  no |
| 8 | f | 58 | PMA, limb onset | 37 | 30 | weakness of legs | yes | no,  felt too sick | no,  no |
| 9 | m | 49 | ALS, limb onset | 42 | 27 | weakness of arm, shoulder and neck muscles | yes | no,  felt too sick, recovering from pneumonia | yes,  no |
| 10 | m | 83 | ALS, respiratory onset | 45 | 42 | weakness of respiratory muscles | yes | no,  felt too tired to communicate, intubated in specialized hospital | yes,  yes |
| 11 | m | 62 | PMA, limb onset | 41 | 36 | weakness of legs | no | yes | yes,  no |
| 12 | m | 49 | PMA, limb onset | 40 | 36 | weakness of legs and bent spine | no | yes,  together with spouse | yes,  no |
| (-) | m | 42 | ALS, limb onset | patient declined participation, he had already participated in 2 pharmaceutical studies and felt too sick | | | | | |
| 13 | m | 39 | ALS, limb onset | 17 | 15 | weakness of arms en legs, dysarthria, dysphagia and weakness of respiratory muscles | no | yes,  with non-invasive ventilation and partly via speech computer,  together with spouse | yes,  still alive |
| 14 | m | 52 | ALS, limb onset | 29 | 25 | weakness of right arm and dysarthria | no | yes,  together with spouse | yes,  no |
| 15 | f | 58 | ALS, limb onset | 41 | 37 | weakness of legs and dysarthria | no | yes,  together with spouse | yes,  yes |
| 16 | f | 67 | ALS, limb onset | 32 | 27 | weakness of right arm, lower legs and hoarseness | no | yes,  together with spouse | no,  no |
| 17 | m | 73 | ALS, limb onset | 45 | 44 | weakness of right arm | no | yes,  together with spouse | yes,  no |
| 18 | f | 61 | ALS, bulbar onset | 30 | 20 | anarthria and dysphagia | no | yes  with speech computer and writing pad,  together with spouse | yes,  yes |
| 19 | m | 41 | ALS, limb onset | 24 | 23 | weakness of arms, neck muscles and dysphagia | no | yes | yes,  yes |
| 20 | f | 54 | ALS, bulbar onset | 32 | 28 | weakness of respiratory muscles, right arm and shoulder | no | yes,  together with spouse | yes,  yes |
| 21 | f | 76 | ALS, limb onset | 37 | 34 | weakness of left arm and shoulder muscles | no | yes,  together with spouse | no,  still alive |
| 22 | f | 59 | PMA, limb onset | 47 | 47 | weakness of left arm | no | yes,  together with spouse | no,  still alive |
| 23 | m | 48 | ALS, limb onset | 32 | 24 | weakness of all limbs and dysarthria | no | yes,  together with best friend | yes,  still alive |
| 24 | m | 65 | ALS, limb onset | 40 | 40 | weakness of left leg | no | yes,  together with spouse | no,  no |
| 25 | m | 36 | ALS, limb onset | 37 | 36 | weakness of arms | no | yes | yes,  still alive |
| 26 | m | 48 | ALS, limb onset | 38 | 33 | weakness of legs and left arm | no | no,  felt too sick | no,  still alive |
| 27 | f | 41 | ALS, limb onset | 30 | 18 | weakness of arms and dysphagia | no | no,  felt too sick | yes,  no |
| 28 | f | 67 | ALS, bulbar onset | 38 | 28 | dysarthria and weakness of all limbs | no | no,  felt too tired to communicate | no,  no |

M = male; f = female; ALS = Amyotrophic Lateral Sclerosis; PMA = Progressive Muscular Atrophy; ALS-FRS = ALS functional rating scale (maximum score: 48; a higher score represents better function retention); (-) = declined participation
